# Gaseous Mercury Exchange from Water–Air Interface in Differently Impacted Freshwater Environments

**DOI:** 10.3390/ijerph19138149

**Published:** 2022-07-02

**Authors:** Federico Floreani, Alessandro Acquavita, Nicolò Barago, Katja Klun, Jadran Faganeli, Stefano Covelli

**Affiliations:** 1 Department of Mathematics & Geosciences, University of Trieste, Via Weiss 2, 34128 Trieste, Italy; nicolo.barago@phd.units.it (N.B.); covelli@units.it (S.C.); 2 Department of Life Sciences, University of Trieste, Via Giorgieri 5, 34127 Trieste, Italy; 3 ARPA FVG Regional Agency for Environmental Protection of Friuli Venezia Giulia, Via Cairoli 14, 33057 Palmanova, Italy; alessandro.acquavita@arpa.fvg.it; 4 Marine Biology Station, National Institute of Biology, Fornace 41, 6330 Piran, Slovenia; katja.klun@nib.si (K.K.); jadran.faganeli@nib.si (J.F.)

**Keywords:** Idrija mercury mine, chlor-alkali plant, mercury evasion, dissolved gaseous mercury, flux chamber, water-air exchange

## Abstract

Gaseous exchanges of mercury (Hg) at the water–air interface in contaminated sites strongly influence its fate in the environment. In this study, diurnal gaseous Hg exchanges were seasonally evaluated by means of a floating flux chamber in two freshwater environments impacted by anthropogenic sources of Hg, specifically historical mining activity (Solkan Reservoir, Slovenia) and the chlor-alkali industry (Torviscosa dockyard, Italy), and in a pristine site, Cavazzo Lake (Italy). The highest fluxes (21.88 ± 11.55 ng m^−2^ h^−1^) were observed at Solkan, coupled with high dissolved gaseous mercury (DGM) and dissolved Hg (THg_D_) concentrations. Conversely, low vertical mixing and saltwater intrusion at Torviscosa limited Hg mobility through the water column, with higher Hg concentrations in the deep layer near the contaminated sediments. Consequently, both DGM and THg_D_ in surface water were generally lower at Torviscosa than at Solkan, resulting in lower fluxes (19.01 ± 12.65 ng m^−2^ h^−1^). However, at this site, evasion may also be limited by high atmospheric Hg levels related to dispersion of emissions from the nearby chlor-alkali plant. Surprisingly, comparable fluxes (15.56 ± 12.78 ng m^−2^ h^−1^) and Hg levels in water were observed at Cavazzo, suggesting a previously unidentified Hg input (atmospheric depositions or local geology). Overall, at all sites the fluxes were higher in the summer and correlated to incident UV radiation and water temperature due to enhanced photo production and diffusivity of DGM, the concentrations of which roughly followed the same seasonal trend.

## 1. Introduction

A key aspect of the Hg biogeochemical cycle is represented by gaseous exchanges between the atmosphere and natural surfaces. In the atmosphere, Hg can persist for more than 1 year due to the high volatility and low solubility of its elemental form (Hg^0^ or GEM, Gaseous Elemental Mercury) [1], which undergoes long-range transport before being converted to the oxidised, more soluble and easily removable form (Hg^2+^) [2,3]. In this way, Hg can reach and impact remote ecosystems far from the points of emission [4]. Direct atmospheric depositions and local supplies deriving from industrial discharge, surface run-off, soil erosion, and leaching are frequently considered the predominant forms of Hg input to freshwater ecosystems [5,6,7,8]. In aquatic environments, both inorganic and organic complexes of Hg^2+^ prevail, depending on pH and redox conditions [9], and their fate is deeply influenced by reactions and transformations involving the Hg^2+^ pool [10]. For example, deposited Hg can be promptly re-emitted to the atmosphere when reduced to Hg^0^ [11,12]. This recycling prolongs the lifespan of Hg in surface reservoirs, enhances its global distribution [13], and, particularly in aquatic ecosystems, contributes to limiting the amount of Hg available for the production/bioaccumulation/biomagnification of the neurotoxic species methylmercury (MeHg) [5,14,15,16]. In natural waters the volatile fraction of Hg, usually referred to as dissolved gaseous mercury (DGM), can account for 1–50% of the total Hg [17,18,19] and is mainly constituted by Hg^0^ produced through abiotic (photochemical) or biotic reduction of Hg^2+^. Photoreduction is considered the dominant process in surface waters [20] where it proceeds at higher rates than biotic reduction mediated by heterotrophic bacteria or algae [21,22]. The high energetic UV radiation (UV-A = 315–400 nm, UV-B = 280–315 nm) is highly effective in promoting DGM production [23,24,25], especially in waterbodies with low levels of dissolved organic carbon (DOC), where a high level of UV penetration through the water column is possible [26,27]. The direct photolysis of Hg^2+^ or the reduction mediated by dissolved organic matter (DOM) are the proposed mechanisms for Hg photoreduction [28], but the role of the latter is still unclear: Mercury complexation by DOM can indeed increase photoreduction by favouring the transfer of solar energy [29], but the formation of strong complexes between Hg^2+^ and reduced sulphur groups [30] can lower the amount of this metal available for photoreactions [31]. In the water column, DGM can be re-oxidised to Hg^2+^ with slower rates than photoreduction [32] mainly through photochemical pathways enhanced by the formation of oxidant radical species [24,33].

When the balance between reduction and oxidation in surface water leads to DGM supersaturation, evasion to the atmosphere can take place [34]. Even though emissions are strongly influenced by the concentration of DGM [35], gaseous Hg evasion to the atmosphere also depends on temperature and turbulent mixing induced by wind [36,37,38].

**Figure 1:**
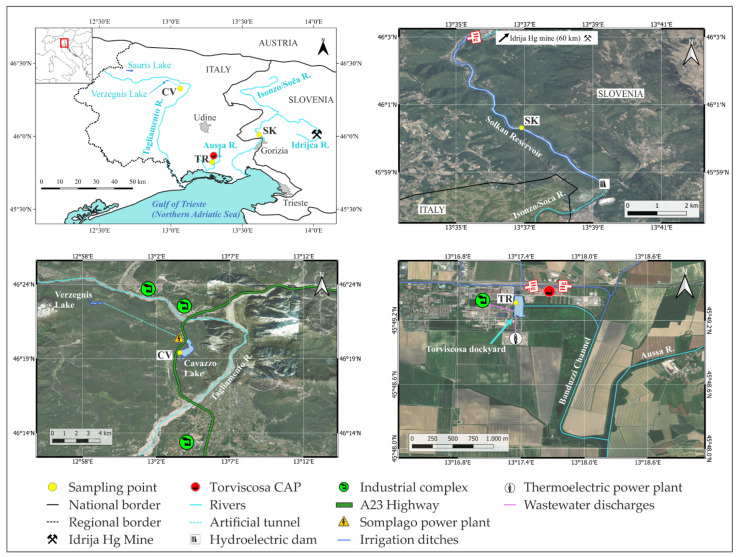
Study area and selected study sites.

The estimate of gaseous Hg exchange from the surface of the water is of paramount importance to assess the fate of Hg in contaminated sites, as legacy emissions will continue to affect its cycling among ecosystems even for centuries [39]. Consequently, the Minamata Convention on Mercury highlighted the need to monitor Hg levels and processes in the environment, with particular focus on contaminated sites [40]. In this framework, the aim of this study was to evaluate gaseous Hg exchanges at the water–air interface (WAI) by means of a floating flux chamber (FC) coupled with a real-time GEM analyser in parallel with total dissolved Hg (THg_D_) and DGM concentrations in two freshwater sites impacted by past anthropogenic activities (mining vs. chlor-alkali industry). In addition, a third site with no known Hg sources was selected as a pristine environment (Figure 1). Sampling was performed during the diurnal period in different seasons to elucidate the variability of the phenomenon in connection with the physico-chemical parameters of water and the meteorological conditions.

## 2. Materials and Methods

### 2.1. Environmental Settings

The Torviscosa dockyard (site TR) is located inside the industrial complex in the low alluvial Friulian plain (NE Italy), the characteristics of which are described elsewhere [41,42]. Industrial activity started in 1937 with the production of cellulose from cane (*Arundo donax* sp.) coupled with a chlor-alkali plant (CAP) using Hg-cells in 1949 [43]. The dockyard extends 380 m N-S and 120 m E-W, with a variable water depth of between 0.3 and 6 m. Freshwater supplies come from numerous irrigation ditches which empty into a main drainage channel, where in the past CAP discharges converged and connected to the northern part of the dockyard. The site is also subject to the influence of the tide from the nearby Marano and Grado lagoon through the Aussa River, with the formation of a “saltwedge” [43]. Moreover, the current sewer drain of the industrial complex and the discharge of cooling waters from the local thermoelectric power plant are located in the southern part of the dockyard [44] and probably influence local water circulation, as a weak surface water current coming from this area was observed in field under ebb tide conditions, whereas a substantial stagnation was encountered during flood tide. The entire area of the complex was subject to the notable input of several contaminants (e.g., PTEs, dioxins, PAHs; [45]) and is currently classified as a contaminated site of national interest following Italian Ministerial Decrees 468/2001 and 222/2012. Mercury contamination is mainly attributable to the past uncontrolled discharges of the CAP: it was estimated that ~186 tons of Hg were discharged into the Aussa River between 1949 and 1984, when a modern wastewater system treatment was installed [43,46]. As a result, extremely high concentrations of this metal are reported for sediments, waters, and air within the industrial complex [47], as well as for the fluvial waters and sediments of the Aussa River [43].

The Isonzo/Soča is a 138 km long alpine river with a catchment area of 3452 km^2^ and represents the main freshwater input for the Gulf of Trieste, northern Adriatic Sea [48]. The hydrological regime is torrential and characterised by maximum flows in April and October/November, and minimum flows in February and August [49]. The course of the river is highly influenced by the presence of hydropower generating dams, which also strongly impacted sediment transport and aquatic ecosystems [50,51]. One of these dams delimits the Solkan artificial reservoir (SK, Figure 1), located about 40 km from the river mouth. The Solkan reservoir was dammed in 1984, has a maximum depth of 20 m, and a length of ~8 km [52]. The mean annual river discharge detected downstream from the reservoir is 80.4 m^3^ s^−1^ [49]. The Isonzo/Soča River represents the main source of Hg into the Gulf of Trieste [53] due to the contaminated material supplied by one of its tributaries, the Idrijca River, which flows through the heavily contaminated historical Idrija Hg mining district [54]. Even though mining ceased in 1996, significant amounts of Hg are still delivered to the Adriatic Sea, mainly in particulate form due to the erosion of contaminated soils, riverbanks, and sediments [55,56], particularly during extreme rain events [57,58]. Covelli et al. [55,57] reported Hg concentrations at the Isonzo/Soča River mouth of 0.46–17.01 ng L^−1^ and of 0.83–112 ng L^−1^ in the dissolved phase and particulate phase, respectively, whereas higher values were found in the Idrijca River waters, particularly during intense rain events (dissolved Hg = 0.57–359 ng L^−1^ [59]; particulate Hg = 0.43–702 ng L^−1^ [56]). As a result of enhanced sedimentation caused by damming, sediments in artificial reservoirs can trap a significant amount of the pollutants transported by rivers [60]; for the Solkan reservoir, Hg concentrations found in sediments range between 5 and 20 mg kg^−1^ [50].

Cavazzo Lake (CV, Figure 1) is a natural freshwater basin located 195 m a.s.l. in the Carnian Alps (NE Italy). It occupies an area of approximately 1.3 km^2^ and has a maximum depth of 38 m in its central part [61,62]. The lake was formed after the Last Glacial Maximum along the palaeochannel of the Tagliamento River in a secondary fluvio-glacial valley carved by glaciers and dammed by end moraine deposits [63,64]. Current natural freshwater supplies are constituted by several seasonally active streams. The lake was subject to notable anthropogenic impacts in the last decades that modified its catchment area: the building of the Somplago hydroelectric power plant, the concurrent digging of an outflow channel (1953–1958), and the construction of a highway viaduct (1973–1979) [62]. The Somplago power plant is fed by an artificial channel that receives water from two upstream artificial basins, Sauris Lake (977 m a.s.l.) and Verzegnis Lake (473 m a.s.l.), and drives cold water through a tunnel into Cavazzo Lake in its northern part [65]. Moreover, this channel strongly increased the sedimentary load to the lake, and sediments deposed after the 1950s show enrichments in Al, Ti, Fe, Sr, S, Zr, Zn, and Pb relative to pristine conditions; this is likely due to the different lithological characteristic of the catchments of the artificial lakes and direct anthropogenic discharges and emissions related to motor vehicle traffic and nearby industrial activities, since two industrial complexes (including plants for wood processing and metal, paper, cement, and steel production) are located within 10 km of the lake [62].

### 2.2. Sampling and Analyses

Gaseous Hg fluxes at the WAI were evaluated during summer (July 2020), autumn (October 2020), and spring (May 2021), while it was not possible to take measurements during winter due to restrictions related to the SARS-CoV2 outbreak. A plexiglass open-bottom floating flux chamber (FC) consisting of one section 50 × 50 × 50 cm, which sits on the surface, and another section 50 × 50 × 30 cm, which is submerged in the water [66,67,68], coupled with a real-time gaseous Hg analyser (Lumex RA915M, Lumex, St. Petersburg, Russia) was used [69]. The instrument facilitates the determination of GEM in the air over a wide range of concentration (from 2 to 30,000 ng m^−3^). Calibration is annually performed by the parent company and checked in the field using an internal reference cell.

Six distinct sets of measurements were taken per day at each study site (from T0 to T5) at regular intervals of 60–90 min. Operatively, the FC was placed on a floating foam board and then manually lowered to the water surface. During sampling, air was drawn through the FC by means of the Lumex internal pump at a constant rate (10 L min^−1^), and GEM concentrations in the headspace were continuously recorded (1 s interval). The adopted flow rate is in the range of those previously used with a similarly shaped flux chamber, ranging between 5 and 20 L min^−1^ in ocean [68] and coastal contaminated environments [66,67], respectively. In this study, the flow rate is lower than those adopted in contaminated settings, since lower Hg concentrations were expected at Cavazzo and reduced flow rates are recommended in this case [70]; however, since a constant flow rate should be used when comparing different locations [71], the value adopted was kept high enough to avoid a potential excessive buildup of gaseous Hg inside the chamber at the sites of Solkan and Torviscosa, which could suppress the emissions. After deployment, the steady state of internal GEM was rapidly achieved (~10 min). At the end of each measure, the chamber was removed from the water, thus limiting its disturbance on the environmental parameters of the surface layer. Moreover, the immersion of the edges of the chamber for 30 cm in water ensures a tight seal, preventing the entry of outside air. Together with the relatively large size of the chamber, this also reduces the influence of other parameters, such as turbulent mixing and waves. However, all measurements were taken under relatively calm conditions, which were optimal for this technique [66]. Gaseous Hg fluxes (F, in ng m^−2^ h^−1^) were then calculated according to the following equation [72]:

(1) $$F=\frac{\left(C_{o}-C_{i}\right)\times \;Q}{A}$$

where Q is the air flow rate through the chamber, A is the surface area of the chamber (0.25 m^2^), and C_o_ − C_i_ is the difference between GEM concentrations in air exiting and entering the chamber (in ng m^−3^). Chamber blanks were checked in the field at the beginning of each sampling day by sealing the FC bottom to a clean polycarbonate surface and they showed negligible values. After each sampling day, the FC was extensively cleaned with diluted laboratory detergent and rinsed several times with MilliQ water.

Atmospheric GEM levels were monitored at the beginning and the end of each sampling day for 20 min using the same sampling interval (1 s) by means of the same analyser; values below the limit of detection (LOD) were set to 1 ng m^−3^ (1/2 LOD) according to the medium bound approach [73].

The intensity of incoming UV radiation in the wavelength range between 250 and 400 nm was monitored in the field by means of a specific sensor (SU-420, Apogee Instruments, Logan, UT, USA) with a resolution of 0.1 W m^−2^. The sensor was installed at ~2 m above ground in unshaded areas close to the sampling points and controlled by means of a laptop computer using Apogee Connect V1.05.003 software (Apogee Instruments, Logan, UT, USA). Data logging was programmed to record data at 1 min intervals as average values of readings were taken continuously every 1 s. Air temperature and relative humidity were also measured in the field using a portable thermohygrometer (HI9565, Hanna Instruments, Padova, Italy).

Temperature, pH, ORP, conductivity, salinity, and dissolved oxygen of surface water were measured in parallel with gaseous Hg fluxes by means of a portable multiprobe meter (HI98194, Hanna Instruments, Padova, Italy). Water samples were collected to determine dissolved organic carbon and gaseous mercury (DOC and DGM), and total dissolved Hg (THg_D_). During summer and spring at site TR, additional water samples taken from the bottom water layer (~2.5 m) were collected using a Niskin bottle.

Water samples for DOC were filtered through pre-combusted (450 °C) Whatman GF/F filters (0.8 μm pore size), collected in glass containers, and frozen until analysis. Analytical determination was performed following a high-temperature catalytic method [74] using a TOC-L Shimadzu analyser calibrated with potassium phthalate and checked via an analysis of certified reference material (Consensus Reference Material, University of Miami, Florida).

DGM was measured following the method described by O’Driscoll et al. [75] on a 1 L fixed volume of water bubbled in a glass container under low light conditions connected with the Lumex analyser in a closed loop circuit. The calculation of DGM (2) was performed on the basis of the equilibrium GEM concentration (GEM_eq_) and the dimensionless Henry’s law constant for Hg (H′) calculated in function of the water temperature [76]:

(2) $$\mathrm{DGM}=\frac{{\mathrm{GEM}}_{\mathrm{eq}}}{H^{\prime }}$$

Samples for THg_D_ were filtered through Millipore Millex HA membrane filters (0.45 μm pore size) into pre-conditioned borosilicate bottles, immediately oxidised by the addition of BrCl (0.5 mL/100 mL sample), and preserved at +4 °C until analysis. Final determinations were conducted according to EPA Method 1631e using the cold vapour atomic fluorescence spectrometry technique (CV-AFS) with a specifically designed detector (Mercur, Analytik Jena, Jena, Germany). The instrument was calibrated using NIST 3133 certificated solution at different dilution levels and characterised by a LOD of 0.63 ng L^−1^ and a limit of quantification (LOQ) of 2.11 ng L^−1^ calculated on the basis of the standard deviations of ten reagent blanks.

Statistical analyses were performed using *R Software 4.1.3* (R Foundation for Statistical Computing, Vienna, Austria [77,78]). The Shapiro–Wilk test [79] was used to test the normal distribution of data and the non-parametric Kruskal-Wallis H test (K-W) to determine whether there were statistically significant differences between two or more groups of an independent variable after testing the normality [80]. As the occurrence of significant differences between data from different seasons was ascertained, Dunn’s post hoc test [81] was performed using the “FSA” R package [82] to identify which groups differ. Finally, the non-parametric Kendall rank correlation coefficient was used to evaluate the associations among variables.

## 3. Results

**Figure 2:**
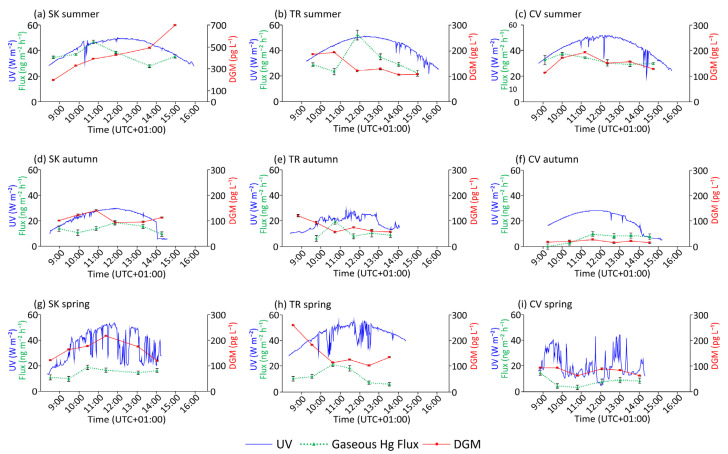
Variation in UV radiation, DGM concentration, and gaseous Hg flux during sampling periods in the different selected sites.

A summary of data collected from all seasons at the selected sites is reported in Tables S1–S3. The variation in gaseous Hg fluxes at the WAI, together with DGM concentrations and incident UV radiation during the sampling periods, are depicted in Figure 2. All sampling campaigns were conducted under conditions of low wind speed and low water turbulence, optimal for the deployment of the flux chamber: hourly data recorded at selected monitoring stations near the sampling points showed that 52% of mean wind speeds during sampling periods were lower than 2 m s^−1^, whereas only one average value was above 5.5 m s^−1^ (“moderate breeze” according to the Beaufort scale, Tables S1–S3). Wind data were provided by the Weather Forecast Regional Observatory of the Friuli Venezia Giulia region (OSMER-ARPA FVG) and the Slovenian Environmental Agency (ARSO) through the database “OMNIA” [83].

### 3.1. Summer

Measurements were performed on days characterised by sunny weather conditions and the absence of clouds, as evidenced by the UV incident radiation patterns (Figure 2a–c). The UV irradiation reached peaks at noon above 50 W m^−2^ at each site. As expected, the highest water temperatures were also observed during this season, slightly higher at TR (range = 19.37–22.90 °C) than the other two sites, where values ranged between 15.96 and 18.68 °C. DOC levels were comparable among all sites, with the highest value recorded at TR (average = 1.5 ± 0.7 mg L^−1^) and the lowest at CV (average = 1.0 ± 0.2 mg L^−1^).

Overall, the highest THg_D_ concentrations in water (Figure S1) were observed at site SK (range = 13.27–32.22 ng L^−1^), which also displayed a strong variability during the sampling period. Both TR and CV showed a diurnal variability characterised by minimum values in the central part of the day and a peak at T5 (TR = 10.66 ng L^−1^, CV = 16.46 ng L^−1^), and the concentrations were, on average, lower than those found at SK. Similarly, DGM concentrations were also higher at SK where an increasing trend was observed during the whole sampling period, from 197.5 to 696.1 pg L^−1^. At TR and CV, the levels of DGM were comparable, although with different trends: in the first case the peaks were found in the morning (T0 and T1) followed by a sharp decrease to relatively constant values in the afternoon; in the second case DGM followed the incoming UV radiation pattern, reaching a peak of 194.0 pg L^−1^ at T2 and then decreasing in the afternoon.

As was the case for THg_D_ and DGM, gaseous Hg evasion fluxes at the WAI were also the highest in summer, ranging on average from 36.65 ± 6.15 ng m^−2^ h^−1^ found at SK to 32.45 ± 3.17 ng m^−2^ h^−1^ at CV, and with the maximum generally recorded at T2, shortly before the peak of radiation, followed by a decrease during the afternoon. At CV, gaseous Hg fluxes showed a smaller variability during the sampling period; in this case, values found in the afternoon were also lower, whereas the maximum was found at T1 in the middle of the morning (10 a.m. local solar time).

Overall, atmospheric GEM showed average values below 3 ng m^−3^, higher at SK (2.77 ± 0.98 ng m^−3^) than TR (1.92 ± 0.95 ng m^−3^) and CV (1.30 ± 0.61 ng m^−3^). Due to a technical issue, at the TR site only the afternoon measurement is available.

### 3.2. Autumn

Optimal weather conditions occurred during autumn sampling at SK and almost all day at CV, where increasing clouds were observed at the end of the sampling period (Figure 2d–f). In both cases, it is notable that the last measures were taken under conditions of reduced irradiation. At TR, there was irregular cloud cover during the day, but it did not significantly affect the absolute value of incident UV radiation; the peaks were comparable at all sites and slightly below 30 W m^−2^, obviously lower than those recorded in summer. As expected, water temperatures were lower than those found in summer at all sites and showed a low diurnal variability (<1 °C). Similar to summer, temperatures were about 2 °C higher at TR than at other sites (range = 13.86–14.87 °C). On average, DOC was higher than that observed in summer at both SK and CV (1.2 ± 0.2 mg L^−1^ and 1.5 ± 0.7 mg L^−1^, respectively), whereas at TR a decrease was observed (average = 1.0 ± 0.3 mg L^−1^).

THg_D_ levels were generally lower than those obtained for summer, especially at TR, where all values were below 2.65 ng L^−1^ (T2) (Figure S1). The highest concentrations were found at SK (maximum of 7.37 ng L^−1^). DGM concentration was also lower than in summer, especially at SK, which is the site where the highest value of this season was recorded (range = 95.9–142.2 pg L^−1^), whereas the lowest concentrations were found at CV (range = 55.8–66.4 pg L^−1^). Here, the diurnal trend was comparable to that found in summer, although with less pronounced variability; the same is true for TR, where after a peak of 122.6 pg L^−1^ at T0, DGM dropped to values comparable to those observed in the lake. Conversely, at SK the diurnal variability was characterised by lower values of DGM after the peak recorded at T2.

Generally, gaseous Hg fluxes at the WAI in autumn were lower than in summer at SK and, in particular, at CV, as confirmed by the average diurnal values of 14.07 ± 3.19 ng m^−2^ h^−1^ and 7.46 ± 2.63 ng m^−2^ h^−1^, respectively. It is notable that no gaseous Hg emission was detected at T0 in CV. In both cases, the diurnal trends of the gaseous Hg fluxes, especially at SK, were characterised by an upward increase to a peak around noon followed by an irregular decrease in the afternoon. Finally, gaseous Hg fluxes calculated for TR showed an irregular variability around values intermediate between SK and CV.

TR was also characterised by a high variability of atmospheric GEM, ranging from <2 ng m^−3^ to 543.61 ng m^−3^, with the maximum detected in the morning. Atmospheric GEM concentrations found at other sites were significantly lower (<3 ng m^−3^ on average) and less variable.

### 3.3. Spring

During May 2021, weather conditions were mostly sunny at TR and variable at SK, but the absolute values of peak UV radiation were comparable to those observed in summer. Unfortunately, sampling at CV was conducted under more extended cloud cover (Figure 2g–i). Water temperatures at SK and CV were comparable to those found in autumn, ranging between 9.35 °C (CV at T5) and 10.48 °C (SK at T3), whereas values measured at TR were higher than the previous season (range = 16.11–16.80 °C). DOC concentration at CV was, on average, close to that observed in autumn (1.5 ± 0.5 mg L^−1^), whereas both SK (average = 0.9 ± 0.3 mg L^−1^) and TR (average = 0.8 ± 0.3 mg L^−1^) were the lowest of all sampling campaigns.

THg_D_ showed a clear variability during the diurnal period (Figure S1), with higher concentrations generally found in the afternoon. Absolute values ranged from <0.63 ng L^−1^ (CV at T1) to 9.96 ng L^−1^ (SK at T4), and only at TR showed an increase compared to autumn. Conversely, DGM concentrations were generally higher than those found in autumn, although they did not reach the levels found during summer, except for the maximum value recorded at TR (259.7 pg L^−1^), which was also the maximum recorded in this season. At SK and CV, DGM followed the UV radiation pattern despite the cloud cover at the latter site, whereas at TR, the trend was the same as other seasons with higher values in the morning.

Generally, the average diurnal gaseous Hg fluxes were of the same order of magnitude as those found in autumn: the lowest fluxes were calculated for CV (8.02 ± 3.96 ng m^−2^ h^−1^), whereas the impacted sites SK and TR were comparable (14.91 ± 3.51 ng m^−2^ h^−1^ and 12.68 ± 6.08 ng m^−2^ h^−1^, respectively). These latter sites also showed a similar variability over the sampling period, with the gaseous Hg evasion peak reached at T2 (between 10:30 and 11:00 local solar time). At site CV, gaseous Hg fluxes followed an opposite diurnal trend, with an initial decrease in the morning followed by an increasing trend in the afternoon.

Atmospheric GEM levels were the highest of all sampling campaigns, with average values of 4.61 ± 4.09 ng m^−3^ at SK and 3.61 ± 2.67 ng m^−3^ at CV. Extremely high values were again obtained at TR in the morning (up to 344.08 ng m^−3^).

## 4. Discussion

Gaseous Hg evasion fluxes at the WAI displayed the highest average value at the Solkan Reservoir (SK, 21.88 ± 11.55 ng m^−2^ h^−1^), which was impacted by historical Hg mining activity. This site was also characterised by both the highest THg_D_ (10.35 ± 8.29 ng L^−1^, range = 2.27–32.22 ng L^−1^) and DGM concentrations (232.6 ± 167.4 pg L^−1^, range = 95.9–696.1 pg L^−1^), which were comparable to those previously reported at the mouth of the Isonzo/Soča River [55,84], but generally lower than those of the Idrijca River [56], which flows through the Idrija mining district. Gaseous Hg evasion was slightly lower at the Torviscosa industrial site (TR, 19.01 ± 12.65 ng m^−2^ h^−1^), where lower DGM levels (125.4 ± 52.9 pg L^−1^, range = 58.1–259.7 pg L^−1^) were found, likely as a result of the reduced availability of THg_D_ in the surface water layer (5.69 ± 3.51 ng L^−1^, range = 1.61–13.68 ng L^−1^), particularly if compared to values previously found downstream in the Aussa River (4.1–52.4 ng L^−1^, [43]). Surprisingly, the gaseous Hg fluxes at Cavazzo Lake (i.e., the pristine area) (CV, 15.56 ± 12.78 ng m^−2^ h^−1^) were similar to those obtained at the other sites, and both DGM (97.7 ± 44.7 pg L^−1^; range = 55.8–194 pg L^−1^) and THg_D_ (5.46 ± 4.51 ng L^−1^, range ≤ 0.63–18.16 ng L^−1^) were comparable to those observed at TR. To our knowledge, Hg supplies to Cavazzo Lake are unknown, thus it can be hypothesised that they may be related to emissions from the industrial complexes located about 10 km and downwind from the lake [85,86,87], but at present no data are available to confirm this hypothesis. In addition, DGM concentrations at CV were also comparable to those found in environments subjected to various anthropogenic sources (e.g., Juam Reservoir = 20–109 pg L^−1^, [85]; Hongfeng Reservoir = 18–109 pg L^−1^, [88]; and Big Dam West Lake = 32.4–182.6 pg L^−1^, [17]), but higher than those commonly reported for background lakes in North America (≤60 pg L^−1^; [22,34,89,90,91]). It should be noted that a geological origin of Hg from the catchment area of the hydroelectrical power plant cannot be excluded: the area is characterised by the presence of Triassic dolostones and limestones with bituminous levels rich in organic matter and carbon intervals [92]. These levels were exploited in the past for coal mining [93,94] and could potentially contain variable amounts of Hg, as reported for other bituminous coal from other areas in the world [95,96]. The increased sediment load to the lake generated by the discharge of the power plant potentially caused the subsequent rise in Hg inputs, as also observed for sulphur and organic carbon [62].

Overall, gaseous Hg fluxes are comparable to or slightly higher than those reported for other freshwater environments (Table 1) subject to Hg supplies from different sources (i.e., domestic and industrial wastewater discharge, atmospheric deposition on the local or long range scale [88,97,98]), but higher than those observed over various natural freshwater systems in North America (e.g., [34,37,99]).

Unfortunately, no measurements were conducted during the night and this could have led to an overestimation of the calculated daily gaseous Hg fluxes [91,97]. In addition, the methods employed for flux measurements add a certain degree of variability. In detail, micrometeorological models generally tend to underestimate the fluxes with respect to flux chamber deployments [17,19,100], but also differences in size, shape, and air turnover time within the chambers used can lead to different results [71]. Thus, our results are directly comparable with those reported using the same experimental approach for the Marano and Grado Lagoon [67], which is downstream from the sites selected in this work and is subject to the same Hg contamination sources (CAP and Hg mining at Idrija). The fluxes found in the lagoon environment were generally higher (range = 11.38–97.38 ng m^−2^ h^−1^), likely due to the more elevated contamination of THg_D_ present in the water column and available for photoreduction to DGM. In our study, weak but statistically significant positive correlations between THg_D_ and DGM concentrations were found both at SK (τ = 0.40, *p* < 0.05) and TR (τ = 0.41, *p* < 0.05), whereas this relationship was not significant at CV (τ = 0.13, *p* = 0.45). Cavazzo Lake was the only site where a negative relationship between THg_D_ and DOC concentrations was observed (τ = −0.43, *p* < 0.05). A possible explanation for these results could be related to an enhanced adsorption of Hg by organic matter and a subsequent reduced availability for photoreduction [28,31]; considering that DOC concentrations were comparable in all investigated sites, this effect may be caused by a different structure of the organic matter, e.g., a reduced content of chromophoric groups or a higher abundance of thiols [24], able to strongly bind Hg [30]. The enrichment of sulphur observed in the lake [62] may support this hypothesis, but further study is needed to better clarify the role of organic matter in Hg photochemistry in this environment.

**Figure 3:**
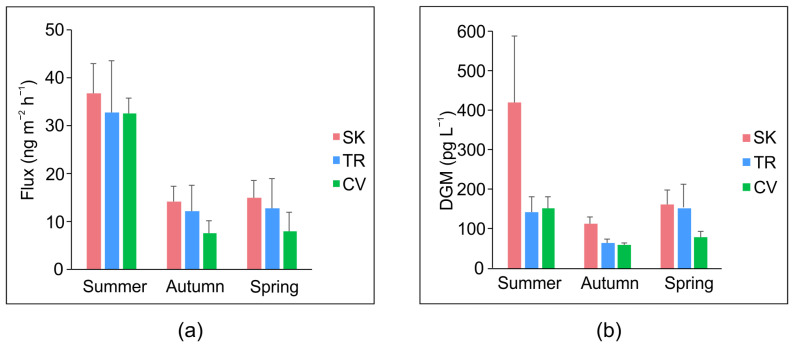
Average diurnal values over the various seasons and selected sampling sites of (**a**) gaseous Hg fluxes, (**b**) DGM concentrations.

Generally, the gaseous Hg fluxes at the WAI were significantly higher in summer (*p* < 0.05, Dunn’s test) than autumn and spring at all sites (Figure 3).

**Figure 4:**
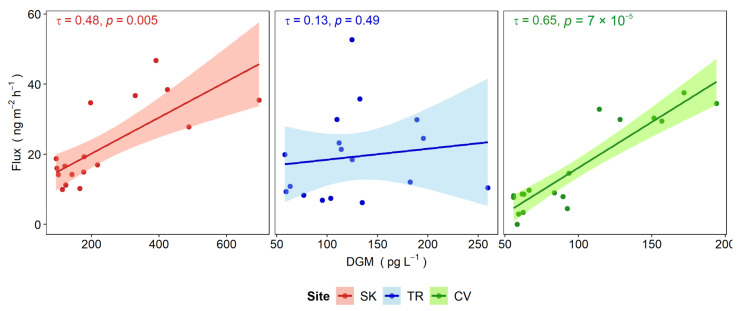
Correlation between DGM concentrations and gaseous Hg fluxes for the selected sampling sites. Kendall’s rank correlation coefficients (τ) and 95% confidence intervals are reported.

The highest values of gaseous Hg fluxes found in summer are related to the intensity of the incident solar radiation, as observed in several studies conducted in both marine (e.g., [67,68,105]) and freshwater environments (e.g., [19,98,99]); in fact, solar radiation is a key factor in promoting a faster rate of DGM production in warmer periods via the photoreduction of Hg^2+^ in surface waters and the subsequent evasion to the atmosphere [26,106,107,108]. This was also confirmed in this study, as high DGM concentrations were detected in summer and the lowest in autumn in parallel with UV radiation intensity, which is most effective in systems with low DOC content that in turn allow for higher light penetration [24,26]. In this work, UV radiation and DGM contents were significantly correlated at both SK (τ = 0.62, *p* < 0.001) and CV (τ = 0.62, *p* < 0.001), but not at TR (Figure S2b) likely due to other atmospheric Hg inputs as discussed below. Similarly, gaseous Hg fluxes at SK and CV were significantly correlated with DGM concentrations (Figure 4), confirming the importance of this latter volatile form in Hg release at the WAI [19], even though it always accounted for less than 10% of THg_D_. This value is in agreement with those usually reported for lake water ([35] and references therein).

**Figure 5:**
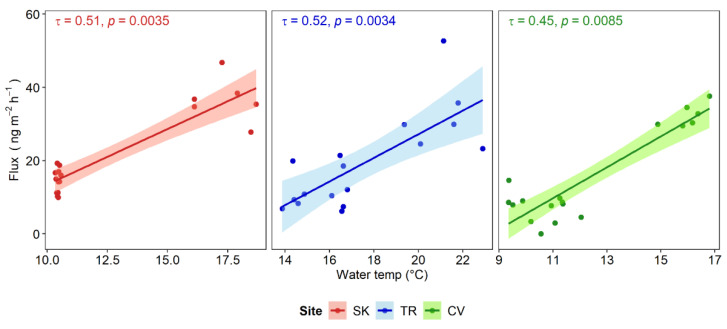
Correlation between water temperature and gaseous Hg fluxes for the selected sampling sites. Kendall’s rank correlation coefficients (τ) and 95% confidence intervals are reported.

As previously mentioned, at site TR no significant correlation between gaseous Hg fluxes and DGM was found, thus suggesting that Hg evasion in this site could be subject to different controls than the amount of DGM [99]. In this site, the strong UV irradiation in spring, with levels similar to summer conditions, potentially enhanced DGM production in addition to low DOC concentration, which could have favoured a higher penetration of radiation [31]. However, the reduced gaseous Hg evasion in spring lies in lower water temperatures compared to summer, as this parameter can significantly affect the equilibrium between water and air, enhancing the solubility of DGM at lower temperatures [98,109]. The positive influence of temperatures on gaseous Hg evasion has been observed in several studies (e.g., [101,103,110]), as was also found in this work (Figure 5).

In addition, gaseous Hg evasion at TR could also be limited by the presence of high atmospheric GEM and by the low dynamicity of the system. During each sampling campaign in this site, the highest DGM concentrations were observed in conjunction with relatively low gaseous Hg evasion and high GEM levels in the atmosphere (>500 ng m^−3^), these latter likely due to the wind driven dispersion of the emissions from the old buildings of the dismissed CAP [47]. High atmospheric GEM can hinder evasion from water surfaces, decreasing the degree of saturation in DGM [34,101], and can also represent a source of Hg for surface water through direct dry depositions [111]. This experimental evidence is supported by the relatively high Hg concentrations found in lichens collected downwind from the CAP [112]. In addition, the water column at site TR suffers from low mixing and thermoaline stratification with the occurrence of a “saltwedge”, as previously observed in the connected Aussa River [43]. The stratification could limit the diffusion of DGM produced at the bottom of the dockyard as the result of dark abiotic and biotic reduction in the bottom water layer and contaminated sediments.

Dark abiotic reduction is mainly a consequence of Hg^2+^ interactions with DOM in the absence of light, particularly with humic substances [113]. The *Mer*-mediated bacterial reduction is catalyzed by Hg^2+^ reductase (Mer A) present in the Hg-resistant bacteria, and is mostly active in oxic environments [114]; the presence of these bacteria was previously reported for the contaminated sediments of the Aussa River [115], nearby our study area. Dark biotic reduction is linked to microbially mediated processes occurring in the dark, including the production and excretion of reducing compounds [116,117], cellular response to oxidative stress [118], DOM mineralisation [119], and unspecific reduction processes [120]. A further contribution to the DGM pool from demethylation reactions could not be excluded; mostly oxidative MeHg demethylation, mediated by sulphate-reducing bacteria producing Hg^2+^, actively occurs in surface sediments of the Marano and Grado Lagoon, along with some reductive demethylation in oxic conditions producing Hg^0^ [121]. Moreover, a pronounced Hg reduction potential in the water column of the nearby Gulf of Trieste, subject to relevant past Hg inputs [53], was demonstrated and assigned most likely to photochemical processes in summer and to phytoplankton (diatoms) and phytoplankton-associated bacterial taxa in autumn [122]. Among them, *Rhodobacteraceaea* and *Gammaproteobacteria* contain known Hg reducers. A recent study by Liang et al. demonstrated that Hg reduction mediated by phytoplankton and algal cell exudates can occur either under sunlit or dark conditions [123].

These processes, together with lower DGM diffusivity in saltwater than in freshwater [38], could explain the higher concentrations detected at the bottom water than in the surface water at the same time during summer at TR; in spring, the salt wedge did not occur, and this phenomenon was less evident (Table S4). In addition, DGM vertical diffusion could also be limited by losses through oxidation under low light conditions [124,125], complexation by chlorides [126], and enhanced flocculation at the salt and freshwater mix zone [55]. The progressive depletion of surface DGM during the sampling periods coupled with increasing evasion fluxes (Figure 2b,e,h) could further suggest limited supplies from deep layers, as observed in other stratified lakes [102]. Further experimental studies are needed to confirm these hypotheses.

Differently from TR, the mining-impacted site of SK, located about 60 km downstream the Hg source, was characterised by lower atmospheric GEM concentrations, which were slightly higher than that of the natural background reported for the Northern Hemisphere (1.5–1.8 ng m^−3^, [127]); this likely caused the surface water to be supersaturated in DGM with respect to the atmosphere over the study period, supporting the observed higher emissions [34]. In conditions of DGM supersaturation, indeed, the rate of gas exchange depends more on the transfer velocity through the interface rather than the occurring gradient concentration [37,38], thus the fluxes can also be influenced by wind speed, water current, turbulence, and turbidity [128]. In conditions of relatively high turbulence, the measures conducted with flux chambers can also be significantly affected [98]. The chamber design adopted in this study limits the influence of these parameters on the flux measured [66] and, in addition, all measures in this study were conducted under relatively calm conditions. However, a possible influence of water movement was detected at site SK, which, differently from TR, was located in a reservoir where the current is actively regulated by an artificial dam. Here, relatively higher DGM concentrations and gaseous Hg evasion occurred when an increase in water flow in the reservoir was observed. Water turbulence could promote both gaseous exchange at the WAI [129] and the DGM supply to the surface layer [67,102]. However, a possible contribution to DGM concentrations at SK related to transport of Hg forms from upstream, a process still ongoing along the Isonzo River both in dissolved form or bound to particulate or organic matter [84], cannot be ignored.

Cavazzo Lake is the only site where DGM concentration and gaseous Hg evasion always follow the incoming UV irradiation relatively well during the sampling period. This could support the hypothesis that Hg present in the area originates from the atmosphere, and it can be readily re-emitted to the atmosphere after deposition. Such recently deposed Hg^2+^ is more available for photoreactions [130] and quickly subject to reduction processes [5,131].

## 5. Conclusions

The formation of DGM and its subsequent volatilisation to the atmosphere are a notable pathway of the Hg biogeochemical cycle, promoting its removal from aquatic environments and thus reducing the pool available for methylation and bioaccumulation. In this context, the estimate of gaseous Hg exchange at the WAI provides useful information on the potential impact of Hg in the environment. In this work, relevant gaseous Hg evasion fluxes were measured in freshwater environments suffering from chlor-alkali industry (TR) and mining (SK) Hg contamination. This was particularly evident in summer when the fluxes were higher than those commonly observed in pristine environments, especially at SK, and suggests that these processes, enhanced by photo-reduction and water temperature, affect the environment even decades after the input of “fresh” Hg. The presence of comparable gaseous Hg fluxes at the pristine site CV suggests that this area should be further investigated for different aspects (i.e., atmospheric depositions, long-term atmospheric measurements, and sediment quality). Other questionable evidence arises at TR, where in spite of the significant contamination, evasion seems to be more affected by atmosphere and water column physico-chemical characteristics. At this site, measurements of benthic fluxes could elucidate the role of sediments as a sink or secondary source of Hg for the water column. In conclusion, the evaluation of gaseous Hg evasion during the nocturnal period would be helpful in reaching a better estimate of Hg budget in these environments, whereas more repeated measurements in each season would likely improve the definition of the pattern of gaseous Hg release during the day.

## Figures and Tables

**Table 1:** Comparison of gaseous Hg fluxes obtained in this study and over various freshwater systems. n.a. = not available, n.s. = not specified.

Measurement Site	Main Hg Source	Gaseous Hg Flux (ng m^−2^ h^−1^)		Method	References
		Mean ± SD	Min–Max		
Solkan Reservoir (SLO)	Hg mining	21.88 ± 11.55	9.96–46.77	DFC	This study
Torviscosa dockyard (ITA)	CAP discharge	19.01 ± 12.65	6.21–52.71	DFC	This study
Lake of Cavazzo (ITA)	Unknown	15.56 ± 12.78	0–37.59	DFC	This study
Baihua Reservoir (CHI)	Organic chemical plant	7.6 ± 2.1	0–50.5	DFC	[98]
Hongfeng Reservoir (CHI)	Atmospheric depositions	5.4 ± 2.3	0.002–36.1	DFC	[88]
Wujiangdu Reservoir (CHI)	Wastewater discharge	-	−11.2–67.2	DFC	[101]
Suofengying Reservoir (CHI)	Wastewater discharge	-	−6.7–23.9	DFC	[101]
Big Dam West (CAN)	Atmospheric depositions	5.4 ± n.a.	0.8–43.8	DFC	[97]
North Cranberry (CAN)	Atmospheric depositions	1.1 ± n.a.	−2.0–13.5	DFC	[97]
Lake Lacawac (USA)	Atmospheric depositions	-	0.14–20.95	DFC	[102]
Puzzle Lake (CAN)	Atmospheric depositions	3.8 ± 2.6	−4.55–9.00	DFC	[17]
Lake Velenje (SLO)	Atmospheric depositions	5.9 ± n.a.	5.3–6.6	DFC	[100]
Lake Ontario (CAN-USA)	n.s.	-	0–9.07	MM	[37]
Lake Michigan (USA)	n.s.	-	0.6–1.6	MM	[34]
Cane Creek Lake (USA)	n.s.	-	0.6–1.2	DFC	[99]
Arbutus Lake (USA)	n.s.	1.6 ± 0.7	-	MM	[91]
Swedish River (SWE)	(Remote area)	11 ± n.a.	−2.5–88.9	DFC	[103]
Lake Gardsjon (SWE)	n.s.	8.5 ± 6.5	-	DFC	[36]
Florida Everglades (USA)	n.s.	1.2 ± 4.9	-	DFC	[104]

## Data Availability

The data presented in this study are available on request from the corresponding author. Meteorological data was obtained from OSMER-ARPA FVG and ARSO and are available upon request at http://www.meteo.fvg.it/ (accessed on 1 March 2022).

## Supplementary Materials

The following supporting information can be downloaded at: https://www.mdpi.com/article/10.3390/ijerph19138149/s1, Table S1: summary of data collected during sampling at Solkan reservoir; Table S2: summary of data collected during sampling at Torviscosa dockyard; Table S3: summary of data collected during sampling at Cavazzo Lake; Figure S1: diurnal variation in THg_D_ concentrations at the selected sites; Figure S2: correlation between incident UV radiation and DGM concentrations; Table S4: parameters in surface and bottom water at TR site in summer and spring.
