# Supplementary material for: Gaseous Mercury Exchange from Water–Air Interface in Differently Impacted Freshwater Environments
Source: Int J Environ Res Public Health. 2022 Jul 2;19(13):8149. doi: 10.3390/ijerph19138149 (PMC9266016; doi:10.3390/ijerph19138149)
Supplement: Supplementary file 1 [file ijerph-19-08149-s001.zip › ijerph-1757455-supplementary.pdf]

Gaseous mercury evasion fluxes from water-air interface in differently impacted freshwater environments

Federico Floreani[1,2]*, Alessandro Acquavita[3], Nicolò Barago[1], Katja Klun[4], Jadran Faganeli[4], Stefano Covelli[1]

SUPPLEMENTARY MATERIAL

Table S1: values of meteorological parameters, DOC, total dissolved and dissolved gaseous Hg concentration, GEM evasion fluxes, and atmospheric GEM levels in the different seasonal samplings at the Solkan reservoir (SK). Wind data provided as hourly averages by Slovenian Environmental Agency (ARSO) through database OMNIA (http://www.meteo.fvg.it/).

	Summer		Autumn		Spring	
	Mean ± SD	Min - Max	Mean ± SD	Min - Max	Mean ± SD	Min - Max
Air temperature (°C)	30.3 ± 2.6	27.2 - 34.5	18.8 ± 3.6	12.8 - 22.4	22.5 ± 3.2	16.5 - 24.8
UV radiation (W m^{-2})	41.8 ± 6.4	27.6 - 49.9	22.6 ± 7.0	5.6 - 29.8	34.3 ± 12.1	13.8 - 53.5
Wind speed (m s^{-1})	2.0 ± 0.8	0.9 – 3.3	2.0 ± 1.1	0.4 – 3.7	1.9 ± 1.1	0.6 – 3.1
Water temperature (°C)	17.42 ± 1.13	16.11 - 18.68	10.49 ± 0.06	10.46 - 10.57	10.41 ± 0.06	10.33 - 10.48
DOC (mg L^{-1})	1.1 ± 0.3	0.8 - 1.4	1.2 ± 0.2	1.1 - 1.4	0.9 ± 0.3	0.7 - 1.5
THg (ng L^{-1})	19.98 ± 7.53	14.31 - 32.22	6.39 ± 0.67	5.51 - 7.37	4.69 ± 2.86	2.27 - 9.96
DGM (pg L^{-1})	421.2 ± 167.0	197.5 - 696.1	112.6 ± 18.0	95.9 - 142.2	163.8 ± 36.8	121.0 - 218.1
% DGM/THg_D	2.4 ± 1.4	0.8 - 4.9	1.8 ± 0.4	1.4 - 2.4	4.2 ± 1.6	1.8 - 6.5
GEM flux (ng m^{-2} h^{-1})	36.65 ± 6.15	34.96 - 46.77	14.07 ± 3.19	9.96 - 18.74	14.91 ± 3.51	10.24 - 19.31
Atmospheric GEM (ng m^{-3})	2.77 ± 0.98	< 2 - 5.28	2.19 ± 2.13	< 2 - 6.48	4.61 ± 4.09	< 2 - 10.62

Table S2: values of meteorological parameters, DOC, total dissolved and dissolved gaseous Hg concentration, GEM evasion fluxes, and atmospheric GEM levels in the different seasonal samplings at the Torviscosa dockyard (TR). Wind data provided as hourly averages by Weather Forecast Regional Observatory of Friuli Venezia Giulia region (OSMER-ARPA FVG) through database OMNIA (http://www.meteo.fvg.it/).

	Summer		Autumn		Spring	
	Mean ± SD	Min - Max	Mean ± SD	Min - Max	Mean ± SD	Min - Max
Air temperature (°C)	31.8 ± 1.5	29.7 - 33.6	16.8 ± 2.1	13.3 - 18.5	23.3 ± 1.2	21.7 - 24.5
UV radiation (W m^{-2})	43.3 ± 7.2	23.0 - 51.7	19.1 ± 5.1	10.3 - 28.6	44.1 ± 6.8	27.2 - 55.2
Wind speed (m s^{-1})	1.7 ± 0.7	0.4 – 2.7	1.0 ± 0.7	0.6 – 2.5	2.9 ± 0.4	1.7 – 3.8
Water temperature (°C)	21.15 ± 1.26	19.37 - 22.90	14.33 ± 0.40	13.86 - 14.87	16.53 ± 0.23	16.11 - 16.80
DOC (mg L^{-1})	1.5 ± 0.7	0.8 - 2.5	1.0 ± 0.3	0.6 - 1.3	0.8 ± 0.3	0.5 - 1.2
THg (ng L^{-1})	9.43 ± 1.22	7.96 - 10.80	1.93 ± 0.37	1.61 -2.65	5.72 ± 2.57	1.89 - 9.12
DGM (pg L^{-1})	143.9 ± 38.5	109.8 - 195.6	79.1 ± 25.5	58.1 - 122.6	153.2 ± 58.9	103.6 - 259.7
% DGM/THg_D	1.6 ± 0.4	1.1 - 2.0	4.3 ± 1.7	2.2 - 6.7	3.3 ± 1.9	1.5 - 5.5
GEM flux (ng m^{-2} h^{-1})	32.68 ± 10.78	23.27 - 52.71	11.07 ± 5.16	6.91 - 15.47	12.68 ± 6.08	9.55 - 35.24
Atmospheric GEM (ng m^{-3})	1.92 ± 2.10	< 2 - 4.61	71.09 ± 98.43	< 2 - 543.61	48.73 ± 77.91	3.38 - 344.08

Table S3: values of meteorological parameters, DOC, total dissolved and dissolved gaseous Hg concentration, GEM evasion fluxes, and atmospheric GEM levels in the different seasonal samplings at Cavazzo Lake (CV). Wind data provided as hourly averages by Weather Forecast Regional Observatory of Friuli Venezia Giulia region (OSMER-ARPA FVG) through database OMNIA (http://www.meteo.fvg.it/).

	Summer		Autumn		Spring	
	Mean ± SD	Min - Max	Mean ± SD	Min - Max	Mean ± SD	Min - Max
Air temperature (°C)	30.4 ± 2.5	25.9 - 32.9	15.7 ± 2.6	12.5 -19.7	17.9 ± 1.7	15.8 - 20.8
UV radiation (W m^{-2})	42.4 ± 7.7	19.3 - 52.2	20.7 ± 7.4	5.3 - 28.2	19.7 ± 9.0	4.9 - 44.5
Wind speed (m s^{-1})	1.9 ± 1.2	0.1 – 3.5	2.1 ± 1.1	1.0 – 3.5	4.6 ± 1.5	2.5 – 6.9
Water temperature (°C)	16.00 ± 0.65	14.88 - 16.80	11.08 ± 0.31	10.55 - 11.36	10.05 ± 1.03	9.35 - 10.18
DOC (mg L^{-1})	1.0 ± 0.2	0.7 - 1.3	1.5 ± 0.7	1.0 - 2.8	1.5 ± 0.5	1.0 - 2.5
THg (ng L^{-1})	9.62 ± 5.34	3.93 - 16.46	3.88 ± 0.57	3.44 - 4.90	2.69 ± 3.03	< 0.63 - 8.49
DGM (pg L^{-1})	152.7 ± 28.9	114.1 - 194.0	59.6 ± 4.0	55.8 - 66.4	80.7 ± 14.5	62.7 - 93.5
% DGM/THg_D	2.3 ± 1.5	0.8 - 4.4	1.6 ± 0.3	1.1 - 1.9	3.2 ± 1.9	0.7 - 5.1
GEM flux (ng m^{-2} h^{-1})	32.45 ± 3.17	29.48 - 37.59	6.22 ± 3.84	0 - 9.79	8.02 ± 3.96	3.41 - 14.63
Atmospheric GEM (ng m^{-3})	1.30 ± 1.00	< 2 - 3.85	1.25 ± 0.62	< 2 - 4.81	3.61 ± 2.67	< 2 - 16.07

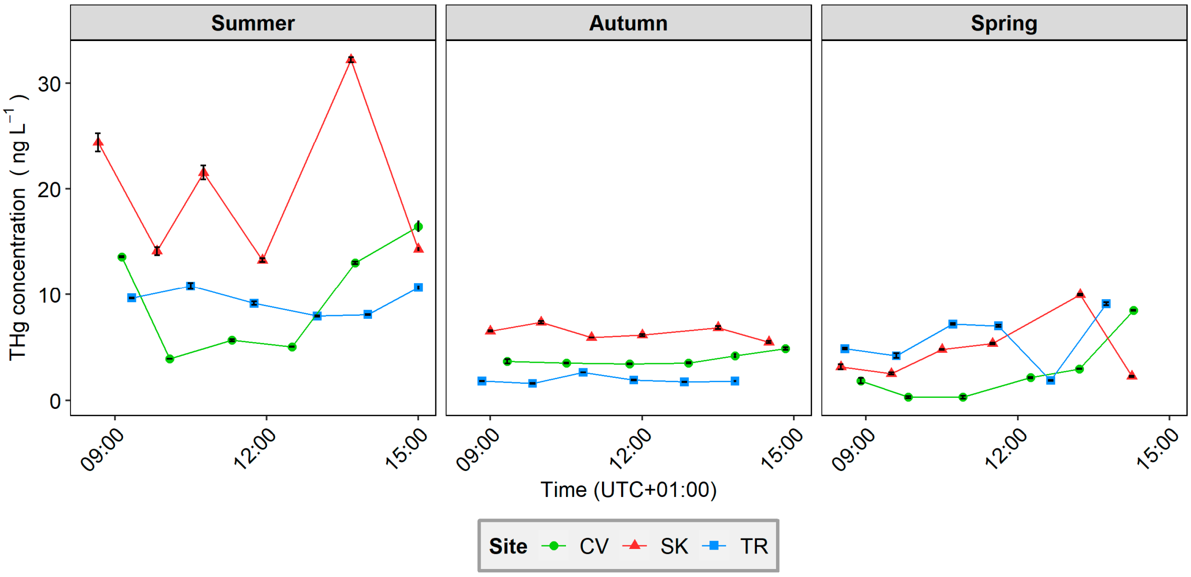

Figure S1: diurnal variation of THg_D concentrations in the different seasons at the various selected sites.

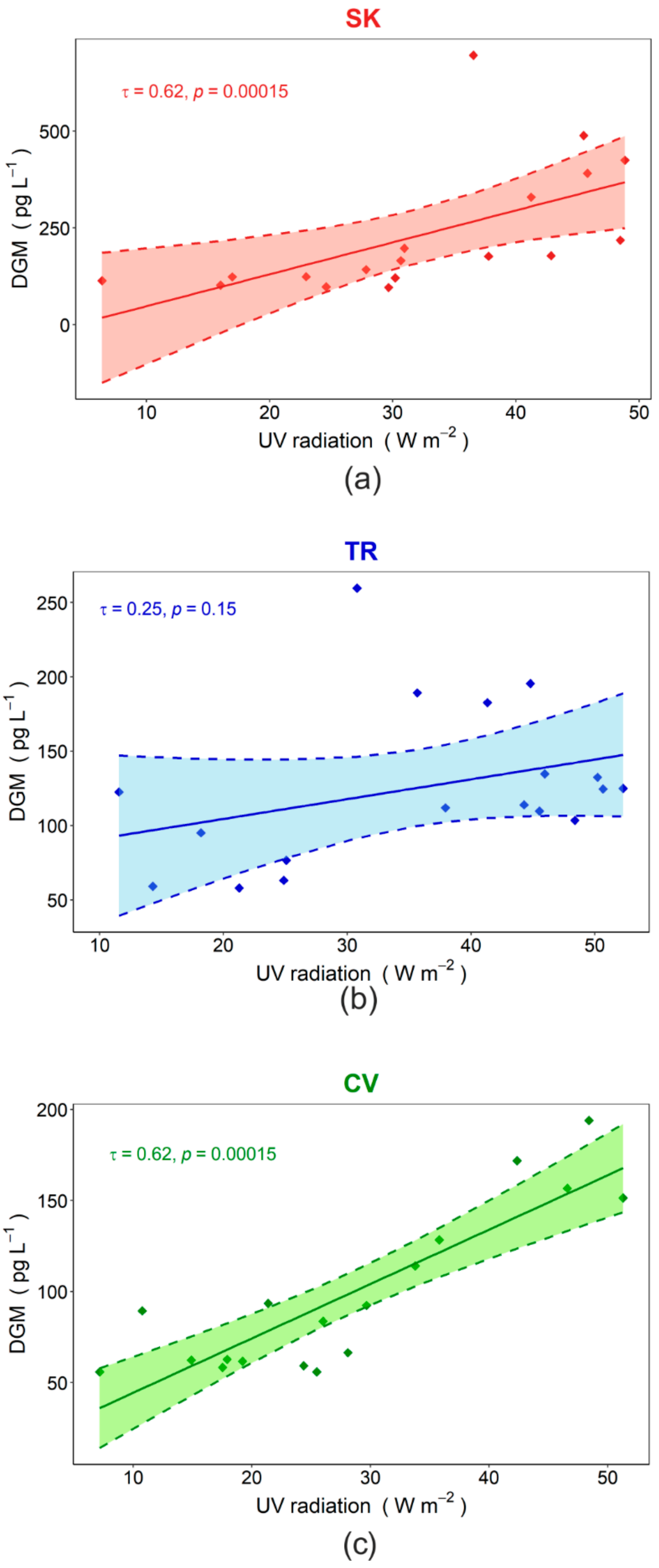

Figure S2: DGM concentrations versus incident UV radiation during samplings at the three selected sites (a): Solkan Reservoir, (b): Torviscosa dockyard, (c): Lake of Cavazzo. Kendall's rank correlation coefficients (τ) and 95% confidence intervals are reported.

Table S4: parameters in surface and bottom water at TR site in summer and spring

	Surface water	Bottom water
28/07/2020-T5 (15:00)		
Temperature (°C)	22.90	21.23
Salinity (PSU)	0.34	12.26
DOC (mg L^{-1})	1.0	7.9
THg (ng L^{-1})	10.66	8.26
DGM (pg L^{-1})	111.9	190.1
10/05/2021-T2 (10:43)		
Temperature (°C)	16.48	17.92
Salinity (PSU)	0.32	0.34
DOC (mg L^{-1})	7.9	1.7
THg (ng L^{-1})	7.20	1.65
DGM (pg L^{-1})	113.9	123.4
